# Genome-scale metabolic modeling of the human gut bacterium *Bacteroides fragilis* strain 638R

**DOI:** 10.1371/journal.pcbi.1011594

**Published:** 2023-10-30

**Authors:** Maxwell Neal, Deepan Thiruppathy, Karsten Zengler

**Affiliations:** 1 Department of Bioengineering, University of California, San Diego, California, United States of America; 2 Department of Pediatrics, University of California, San Diego, California, United States of America; 3 Center for Microbiome Innovation, University of California, San Diego, California, United States of America; National Institute for Public Health and the Environment, NETHERLANDS

## Abstract

*Bacteroides fragilis* is a universal member of the dominant commensal gut phylum Bacteroidetes. Its fermentation products and abundance have been linked to obesity, inflammatory bowel disease, and other disorders through its effects on host metabolic regulation and the immune system. As of yet, there has been no curated systems-level characterization of *B*. *fragilis*’ metabolism that provides a comprehensive analysis of the link between human diet and *B*. *fragilis*’ metabolic products. To address this, we developed a genome-scale metabolic model of *B*. *fragilis* strain 638R. The model *i*MN674 contains 1,634 reactions, 1,362 metabolites, three compartments, and reflects the strain’s ability to utilize 142 metabolites. Predictions made with this model include its growth rate and efficiency on these substrates, the amounts of each fermentation product it produces under different conditions, and gene essentiality for each biomass component. The model highlights and resolves gaps in knowledge of *B*. *fragilis*’ carbohydrate metabolism and its corresponding transport proteins. This high quality model provides the basis for rational prediction of *B*. *fragilis*’ metabolic interactions with its environment and its host.

## Author summary

The bacteria within our digestive system play many critical roles in linking what we consume to our health. They can break down compounds we cannot, making more nutrients available. Moreover, the byproducts they produce can influence the human body to make different choices, such as storing more or less energy as fat or raising blood sugar levels. There is therefore a need to understand and predict which byproducts the major gut bacteria will produce when given different nutrients, which would expand our understanding of the connection between diet and metabolic disorders like diabetes and inflammatory bowel disease. To this end, we constructed a mathematical model of the metabolism of the common gut bacterium *Bacteroides fragilis*. With this model, we outlined the bacterium’s ability to use 142 different nutrients and the range of possible byproducts. We further analyzed how these nutrients would impact the ratios of the most prominent byproducts, examined how it could digest the complex sugars found in milk, and predicted that it could use the byproducts of other bacteria. Altogether, our results outline the possible roles *B*. *fragilis* plays in the human gut and begin to determine how it fits into the broader community of bacteria around it.

## Introduction and background

*Bacteroides fragilis* is a human commensal colon bacterium [1]. It is gram-negative, anaerobic but somewhat aerotolerant, and ferments dietary fibers and resistant starches [1,2]. The bacterium belongs to the phylum Bacteroidetes, which along with Firmicutes comprises 90% of the total gut microbiota [3], and is essentially universal in humans. *B*. *fragilis* can consume an assortment of carbohydrates, including host protein glycans [1,4]. The bacterium excretes volatile fatty acids (VFA) and other carboxylic fermentation products such as acetate, propionate, lactate, and fumarate. The regulation of liver and intestinal human metabolism is partially influenced by these products and their abundance ratios. These metabolites are therefore associated with the development of obesity, inflammatory bowel disease (Crohn’s disease and ulcerative colitis), and other diet associated disorders [5–8]. The metabolic associations are different for each of these fermentation products. Acetate can suppress appetite, reducing overall caloric intake. Propionate is known to inhibit lipogenesis, while acetate promotes it, in certain conditions. Both propionate and butyrate promote gluconeogenesis, affecting blood sugar homeostasis [6,8]. Thus, while carbohydrates promote overall VFA production in the gut, the effects on host metabolism vary significantly depending on the metabolic activity and fermentation profiles of specific bacteria. *Bacteroides* species primarily produce acetate and propionate, while members of the Firmicutes phylum produce more butyrate [1,5].

While the metabolism of the related *B*. *thetaiotaomicron* and its carbon utilization has been well studied and mathematically modeled [9], our knowledge of *B*. *fragilis*’ metabolism has been relatively limited. Therefore, the ability to predict the production flux ranges of these VFAs from the ubiquitous *B*. *fragilis* and its growth rates in various environments is critical for delineating *B*. *fragilis*’ and the other *Bacteroides* species contributions on host physiology.

Current efforts have been faced with issues connecting individual observations about growth phenotypes or enzyme activities to form a complete understanding of *B*. *fragilis*’ metabolism and design strategies to modulate its behavior. Genome-scale metabolic models (GEMs) overcome this barrier by representing metabolism with a set of reactions derived from the organism’s genome [10]. Therefore, a GEM would create a comprehensive, quantitative model of metabolism, enabling the user to elucidate the interactions between *B*. *fragilis*’ metabolic components and optimize VFA production rates [11].

An annotated genome provides the GEM with enzymes and transporters that are available to the organism and associates them with biochemical reactions. During the manual curation of the GEM, enzyme functions and transporters may be added and corrected with available data in the literature. Manual curation is further supported by databases such as BiGG [12] and KEGG [13], which contain information on enzymes, reactions, and substrates for the purposes of metabolic modeling and analysis. Then lipidomics, proteomics, and other data are integrated into the GEM to describe the metabolites needed to produce the organism’s biomass, which yields the biomass reaction [14].

Furthermore, the metabolic rates in a GEM are constrained by the definition of the available resources in the media and by the reversibility of all reactions as inferred by thermodynamics. These constraints, assumptions, and reaction stoichiometries narrow down the possible ranges of metabolic reaction fluxes. Therefore, a GEM is able to identify which reactions and pathways must be active and at what rates under defined conditions. To date, GEMs have been used to predict the growth rate or ability to utilize a variety of nutrients, the maximal output rates of desired metabolites, and the minimal number of reactions needed to produce a desired output for thousands of species across all domains of life [12,15].

In this work, we reconstructed a GEM for *Bacteroides fragilis* strain 638R refined with experimental growth and metabolite utilization data. We subsequently deployed the model to analyze systems-level features of its metabolic network, its ability to produce different VFAs across conditions, and its efficiency in utilizing different carbon sources for growth. Together, our results outline *B*. *fragilis*’ potential metabolic role within the gut microbial community by identifying its possible inputs, outputs, and preferred substrates. We also compare our model to an existing semi-automatic reconstruction. The model, named *i*MN674 to follow convention, enables the integration of multi-omics data sets and the generation of hypotheses with respect to the metabolism and niches of *B*. *fragilis*.

## Results and discussion

### Model reconstruction

**Fig 1:**
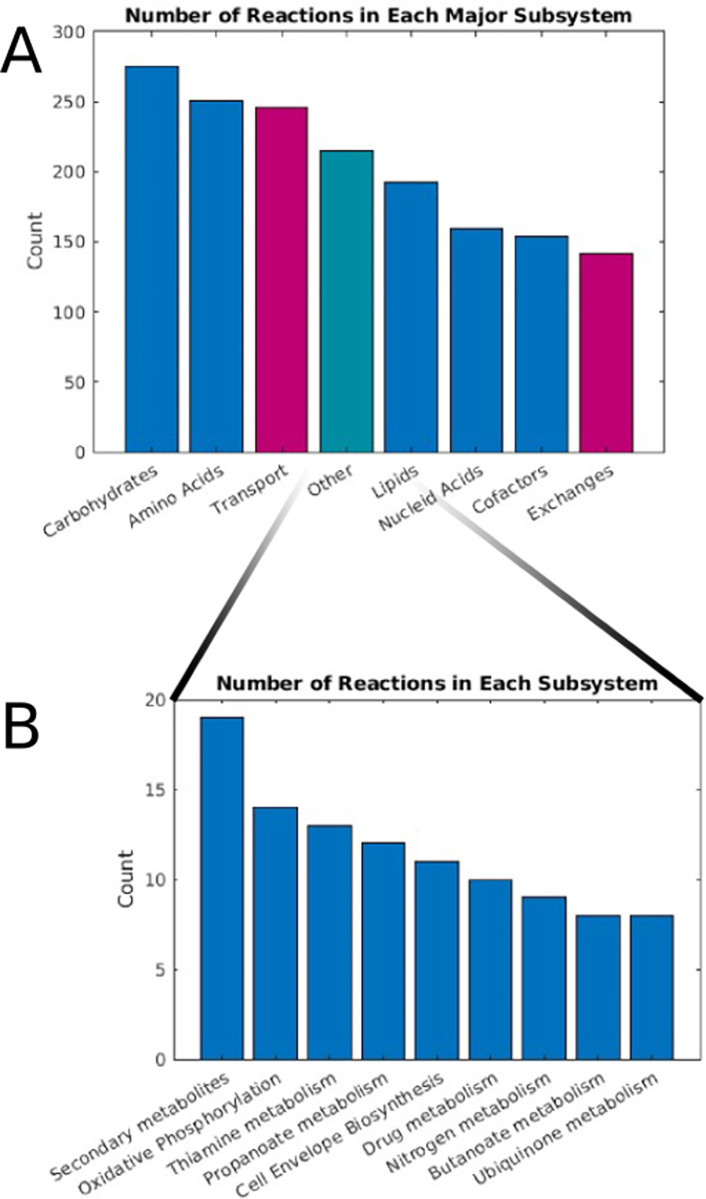
An overview of the model’s reactions and basic properties. (A) In the major subsystem plot, subsystems associated with major macromolecule types were grouped. Transport associated reactions are shown in red. (B) The secondary subsystem bar plot, which expands the “other” category in the first plot.

The genome-scale metabolic reconstruction *i*MN674 of *Bacteroides fragilis* contains 1,109 metabolites distributed across three compartments, i.e. the extracellular space, the periplasm, and the cytosol. The model contains 1,362 metabolites in total associated with 1,634 reactions. The associated pathways of these are broken down in Fig 1. Of these reactions, 142 are exchange reactions, 253 are transporters, and the remaining 1,239 are metabolic. The reactions are associated with 674 genes excluding pseudogenes identifying non-enzymatic reactions, exchanges, and reactions with no identified gene. The complete model is available as a MATLAB file, COBRApy json file, and spreadsheet format (S1 and S2 Files).

Our model was generated following the procedure outlined by Thiele and Palsson [10], beginning with finding reactions associated with genes in the annotated genome on the KEGG database. Additional pathways not present in the database were gathered through a search of the literature (see the “Reference” column of the reactions sheet of S2 File for reactions added this way). The initial draft model was assembled using these gathered reactions, the necessary exchange reactions to provide metabolites, and an initial biomass composition estimate. This is followed by an iterative process of examining the model’s growth on known substrates, accuracy of ATP production, and other features. Deficits are fixed by delving back into the literature and finding additional transporters, reactions, and constraints to further refine the model.

Nine reactions were included in the model without genome annotation, excluding non-enzymatic reactions. For comparison, *i*ML1515, a highly comprehensive model of *E*. *coli*, contains 113 such reactions [16]. In *i*MN674, three of these were transporters for metabolites *B*. *fragilis* was experimentally shown to consume, but no suitable genes could be found to import these metabolites. Transport mechanisms in the Bacteroidetes phylum as a whole remain unclear for a number of carbohydrates and further work will be required to elucidate these processes [1]. In addition to missing annotation for genes involved in carbon utilization, there is also a lack of information on the genes required for replication. About half of the essential genes in *B*. *fragilis* 638R previously determined through transposon gene disruption have no identified function [17]. Our model has a sensitivty and specificity of 0.27 and 0.86 when predicting gene essentiality. Note that as so many essential genes lack annotation, they could not be included in the mode. S1 Table contains this comparison and a list of essential genes. One reaction in *i*MN674 with no gene association is thymidine monophosphate kinase (E.C. 2.7.4.9). This reaction is necessary for the production of dTDP from dTMP, and thereby the production of dTTP for DNA replication. No match for this enzyme in *B*. *fragilis*’ genome could be identified. The enzymes on either side of this reaction in the dTTP production pathway are present, so in consideration of the necessity of this enzyme for growth the reaction was included in the model without a gene association. A complete list of reactions without genome annotations is provided in S2 Table.

These unknowns and gaps leave 25 percent of the reactions unable to carry flux. These reactions are generally associated with secondary metabolites and are entirely uninvolved in producing growth metabolites. For example, the genome contains several enzymes involved in the production of quorum sensing molecules, but the complete pathways have not been identified [18]. This renders the few known quorum sensing reactions disconnected from the metabolic network. There are also reactions associated with the degradation and export of various drugs. As these miscellaneous molecules are not otherwise part of the model, their associated reactions cannot carry flux. Many such reactions are secondary uses of more common enzymes which are added to automated reconstructions in a species independent manner. Additionally, this may also be a reflection of *B*. *fragilis*’ apparent proclivity to carry a diverse set of enzymes for a broader range of possibilities [1].

**Fig 2:**
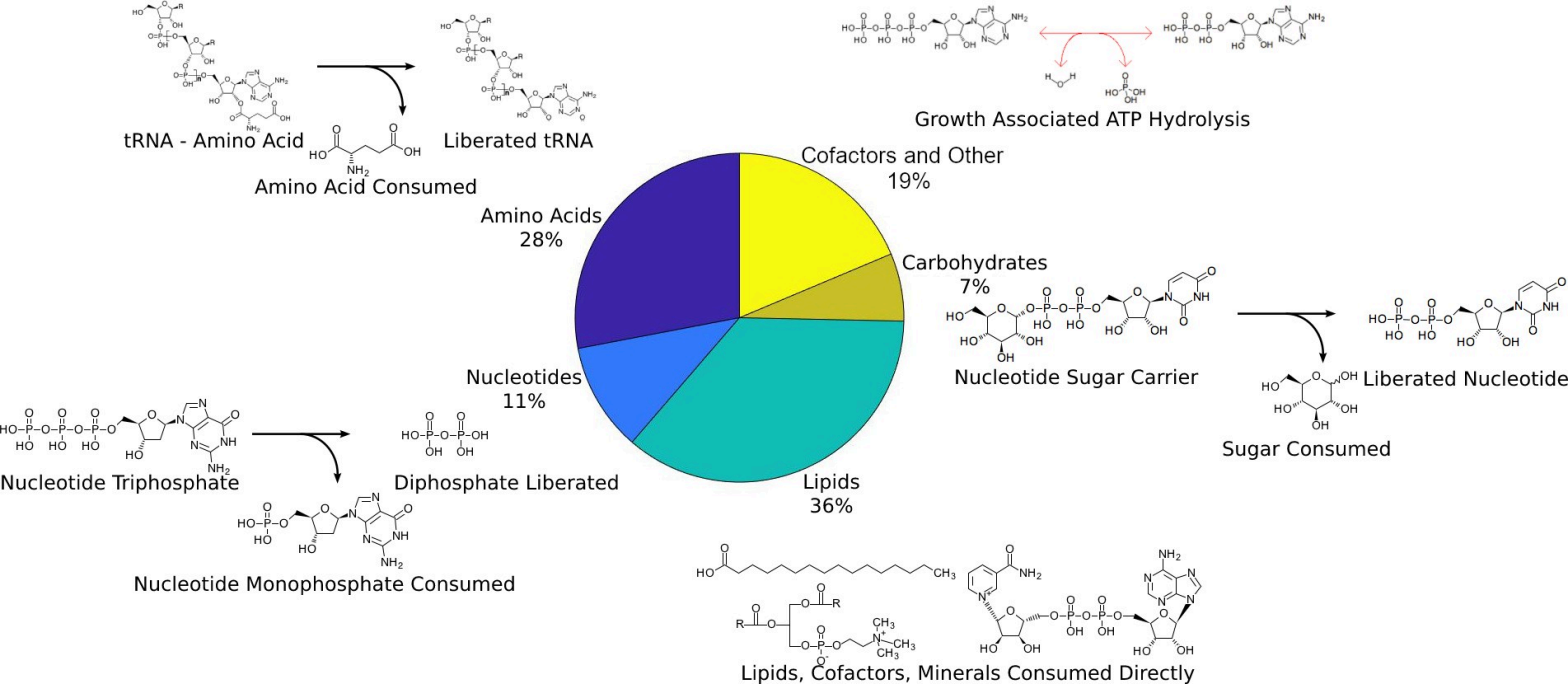
The biomass components consumed in the model. The central pie chart shows the percent of consumed metabolites that belong to each category. Next to each sector is a diagram representing how a typical component is represented in the biomass reaction. Water, hydrogen, and other minor components of these reactions are not shown.

The biomass reaction provides the objective function in most simulations performed and its components define which pathways must be active under any growth conditions [10]. We defined a biomass reaction consisting of amino acids, lipids, carbohydrates, DNA and RNA monomers, cofactors, minerals, and the cycling of carrier molecules for these components like tRNAs. This information was compiled from data on *B*. *fragilis* and other members of the *Bacteroides* genus (see Methods - Biomass objective). The biomass reaction contains 98 metabolites, and is as detailed as possible to maximize the model’s accuracy. Its components are summarized in Fig 2.

### Experimental nutrient utilization

We validated *B*. *fragilis* 638R’s ability to utilize 44 nutrients in a dilute complex medium experimentally. The change in OD600 after two days was measured to determine which nutrients *B*. *fragilis* utilizes (see Methods - Nutrient utilization experiment*)*. Then the model was simulated with and without each nutrient to confirm if the model also predicted an increase in growth rate. The model predicted that many metabolites would only be beneficial in conjunction with others, as elaborated upon in Results—Co-metabolism. Thus a dilute complex medium was chosen to reveal which nutrients could be utilized, without the need of testing thousands of combinations.

**Fig 3:**
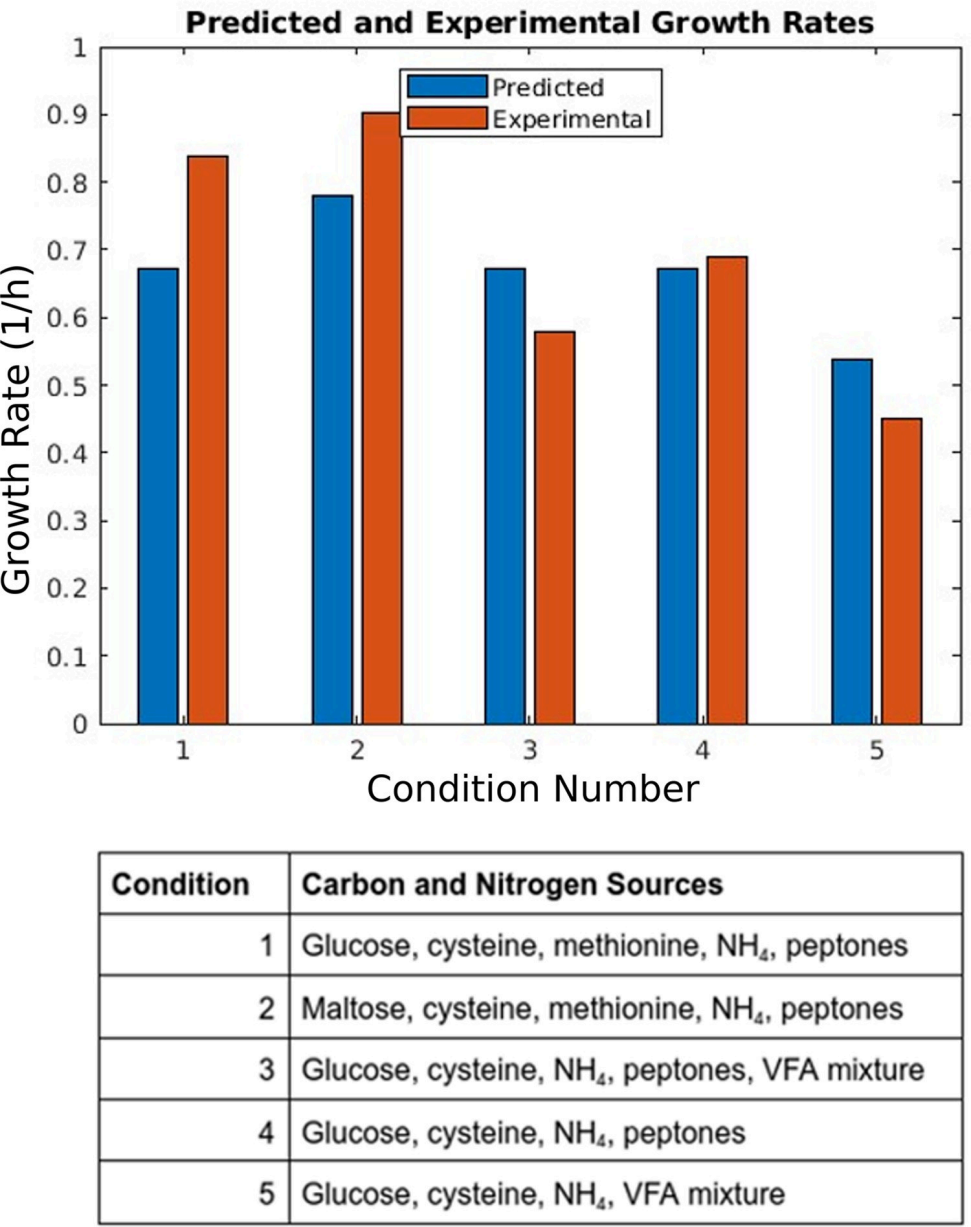
A comparison of *in silico* and experimental growth phenotypes. This bar chart compares predicted and experimental growth rates for five media from the literature. The legend below describes the carbon and nitrogen sources, with numbers corresponding to the bars in the main figure. Full details are present in S4 Table. Conditions 1 and 2 were found in Spence *et al* [19], Fig 2A and 2B. Conditions 3–5 are from Varel and Bryant [20], Fig 1 curves 1, 3, and 4. For most metabolites, the associated exchange reaction was allowed a maximal uptake of 20 millimoles per gram dry weight of organism per hour (mmol/gDW/h), except maltose which was constrained to 10 mmol/gDW/h as it contains two units of glucose.

The results of our growth experiment and other growth predictions are summarized in Fig 3. Our model achieves a sensitivity and specificity of 0.9 and 0.96 when predicting growth or no growth on these nutrients. The full results are in S3 Table. Full simulation details are in S4 Table.

Additionally, the model was curated to predict growth phenotypes in a variety of media routinely used to grow *B*. *fragilis* [19–23]. The ATP requirements of the model were optimized to constrain the growth rate within 20% of the values presented in Varel and Bryant and Spence *et al* [19,20], those used in Fig 3. This yielded a growth-associated ATP maintenance (GAM) coefficient of 24.9 and a non-growth (NGAM) coefficient of 38.9. Our GAM is significantly lower than the values in many other models (such as 75.5 in iML1515), due to us explicitly modeling ATP consumption in tRNA charging and amino sugar synthesis for biomass (see Methods - ATP maintenance coefficients). The mean percent error over the data from these sources was 14%. *B*. *fragilis* cannot use amino acids as the sole nitrogen source [20], and we found no growth improvement in the dilute complex medium when amino acids were added.

With the incorporation of these growth rates from the literature and our metabolite utilization experiment, we have consolidated the available growth rate data and expanded the knowledge of which substrates *B*. *fragilis* may use. Subsequently, we analyzed carbon utilization pathways and determined condition-dependent fermentation profiles.

### VFA production

The model captures the production of a variety of VFAs from glucose or other carbon sources. The fermentation products identified in Onderdonk and Gorbach and Frantz and McCallum (1979) [22,24] were examined to determine how much of each VFA could be produced at a fixed growth rate and carbon intake. Isovalerate was not included in the model, as the pathway that produces it has not been fully characterized. Table 1 displays these results.

**Table 1:** Fermentation Product Output Rates (mmol/gDW/h) on a Minimal Glucose Medium at a Fixed Growth Rate.

	Ethanol	Acetate	Propionate	Butyrate	Succinate	Fumarate	D-Lactate	L-Lactate	CO2
Ethanol	**25.5**	8.17	0	0	0	0	0	0	26.49
Acetate	0	**46.92**	0	0	0	0	0	0	0
Propionate	0	13.22	**19.08**	0	0	0	0	0	10.14
Butyrate	0	46.43	0	**0**	0	0	0	0	0.98
Succinate	0	27.86	0	0	**9.53**	0	0	0	0
Fumarate	5.63	28.03	0	0	0	**6.62**	0	0	0
D-Lactate	0	8.17	0	0	0	0	**25.5**	0	0.98
L-Lactate	0	8.17	0	0	0	0	0	**25.5**	0.98
CO2	25.50	8.17	0	0	0	0	0		**26.49**

To construct Table 1, the model was simulated with glucose, ammonia, sulfide, and minerals (see S4 Table). The growth rate was constrained to be at 95% of the optimal value, in the range [0.406, 0.428]. This allows for a broader range of metabolic choices to be represented rather than being limited to the one optimum. The model was then optimized to maximize the output of each metabolite along the rows. The corresponding column is the amount of each carbon containing product produced. Thus, row one represents the results of the simulation when ethanol production was the objective being maximized. The rows of this table thereby outline the range of possible fermentation product outputs. The main diagonal represents the maximum amount of each that may be produced.

In each of the cases shown in Table 1, the model predicts that of the 120 mmol/gDW/h of carbon entering the system as 20 mmol/gDW/h of glucose, 93.8 mmol/gDW/h or 78% of the original carbon leaves the system as one of the above fermentation products or carbon dioxide; the rest becomes biomass. The VFA ratios in Frantz and McCallum (1979) [22] are attainable by the model without altering the optimal growth rate by more than 5%. These results outline the range of VFA production possibilities; any values between those shown in the table will also be possible. The actual *in vivo* ratios of output products must be determined by factors other than which pathways are available. These could include transcriptional control, translational control, or enzyme inhibition.

The model predicts that acetate and propionate are partially decoupled. Acetate can be produced without propionate, but propionate production is acetate dependent. In general, the model primarily produced acetate and there were no conditions in which acetate flux was zero. The model could not produce butyrate at all, in line with the observed phenotype for this species. There are a few butyrate-utilizing enzymes present in the model, but the production pathway is incomplete. A similar result has been previously observed in the reconstruction of *B*. *thetaiotaomicron*’s metabolism [9].

Additionally, the model was analyzed to determine which metabolite would allow for the largest increase in the production of each fermentation product. When added to provide an additional flux of 10 mmol carbon per gram dry weight per hour (C-mmol/gDW/h), maltose provided the greatest increase in yield of all of the products except butyrate. The acetate to propionate ratio was most decreased by glucose and other carbohydrates. It was most increased by supplementation of amino acids and glucosamine. In general, nitrogenous compounds increased the acetate to propionate ratio while simple carbohydrates decreased the ratio. S5 Table contains the full results. This implies that propionate production increases most in nitrogen depleted, carbohydrate rich conditions, such as a low protein or high carbohydrate diet [25].

### Growth efficiency on different carbon sources

**Fig 4:**
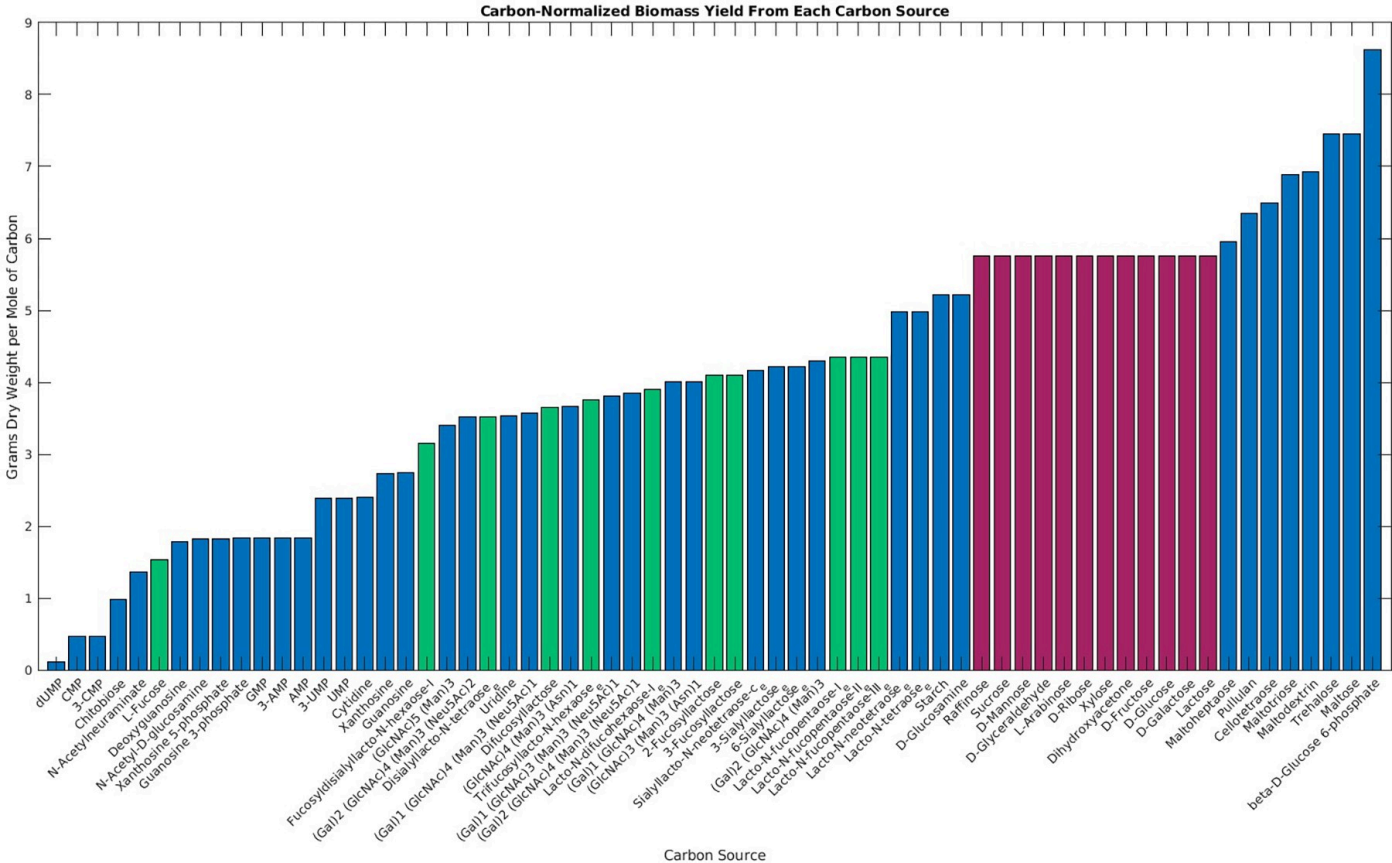
Simulated growth yields per mole of carbon on different substrates. *i*MN674 was simulated on a minimal medium containing ammonia, sulfide, and minerals. The carbon source indicated in each bar was given a maximal intake flux of 120 C-mmol/gDW/h. The predicted growth rate was divided by the carbon flux to indicate the growth efficiency. Metabolites in green contain fucose. Metabolites in red have the same efficiency as glucose.

To predict which metabolites best promote growth of *B*. *fragilis*, the efficiency of *B*. *fragilis*’ growth on various carbon sources was determined (Fig 4). This efficiency, calculated as simulated biomass yield per millimole of carbon taken in, represents how well a given carbon source can be incorporated into biomass. Common carbohydrates like glucose, starch, and fructose are the most efficiently used by *B*. *fragilis*. Some of these can either be immediately hydrolyzed to glucose or are converted to glucose in the non-oxidative pentose phosphate pathway. Therefore, their efficiency is similar to that of glucose. On the lower end of the efficiency spectrum is fucose, which was predicted to have 26% the efficiency of glucose. Fucose is split into two molecules, dihydroxyacetone phosphate and L-lactaldehyde. The former is transformed into pyruvate via glycolysis, while the latter is converted into propanediol and exported, producing no further energy. Fucose-containing substrates such as 2’- and 3’-fucosyllactose similarly showed a reduced efficiency. These and other fucosylated oligosaccharides form 35–50% of human milk oligosaccharides [26]. The inefficiency of *B*. *fragilis* in metabolizing fucose-containing carbon sources may help explain why *B*. *fragilis* and other Bacteroidetes species are less common in the neonatal gut microbiome than in adults [27].

Two of the most efficient substrates identified by the simulation are maltose and glucose-6-phosphate. The maltose phosphorylase reaction splits the maltose units apart and yields glucose and glucose-1-phosphate. This can be converted to glucose 6-phosphate via phosphoglucomutase. Therefore, half of the carbohydrate in maltose enters glycolysis without ATP use, greatly improving the energy yield. Starch is instead hydrolyzed into glucose subunits and does not provide this benefit.

### Co-metabolism of fermentation products and amino acids

While discussion of *Bacteroides’* metabolism is typically focused on carbohydrates, amino acids and fermentation products from other microbes are generally available to these microorganisms in the colon. *B*. *fragilis* cannot utilize any single amino acid or mixture of amino acids as the sole nitrogen source [20] and the model reflects this. Our model further suggests that *B*. *fragilis* cannot produce ATP from acetate, propionate, butyrate, or ethanol. However, the presence of these fermentation end products in addition to other substrates still confers a growth advantage for *B*. *fragilis*. It has been previously observed that *B*. *fragilis* consumes acetate in conjunction with other organic molecules and is enriched by the consumption of alcohol, which is converted to acetate by the liver [28]. To investigate the potential for VFA co-metabolism, the model was simulated with a range of carbon sources in addition to a mixture of fermentation products, a mixture of amino acids, both mixtures, or neither, all provided at a flux of up to 20 mmol/gDW/h. The growth rates under these conditions were then compared. The addition of these molecules was considered to improve the growth rate if the rate increased by more than 0.001 h^-1^. Conditions where the model predicted growth with VFAs or amino acids are listed in Table 2.

**Table 2:** Predicted Growth Rates for Metabolites Which Were Not a Sole Carbon Source, but Allowed Growth When Supplemented with VFAs or Amino Acids.

Metabolite	Growth with VFAs (h ^-1^)	Growth with Amino Acids
Acetaldehyde	0	0.11
Alanine	0.09	0
Aspartate	0.11	0
Citrate	0.32	0.11
Ethanol	0	0.11
Fumarate	0.11	0
Glutamate	0.1	0
Lactate	0.1	0.11
Malate	0.32	0.11
Succinate	0.11	0

The VFA mixture, containing acetate, propionate, butyrate, ethanol, and pyruvate, did little to improve the growth rates of *B*. *fragilis*. When it did improve, it increased by only 6% on average, despite the massive increase in available carbon. The presence of fermentation products allowed for weak growth on lactate, malate, fumarate, succinate, and several amino acids, none of which could act as sole carbon sources. Generally, the VFAs were incorporated into acyl-CoA and used to assemble lipids. Since VFAs can not be used to produce the same range of biomass precursors as glucose, utilization of VFAs only marginally improved the growth rate of *B*. *fragilis*.

The amino acid mixture had a greater impact on *B*. *fragilis’* growth and resulted in a 15% increase in growth on average across conditions. This included a 30% increase in growth on glucose and glucose equivalents. In addition to improved growth in the presence of amino acids, we also observed a greater resilience of *B*. *fragilis* to gene knockouts. When glucose was the sole carbon source, 127 genes were essential for producing at least one biomass component. These genes were identified by deleting each gene and its associated reactions in the model one at a time to simulate single gene knockouts. If the model predicted no growth, the gene was labeled as essential. Each of these genes, on average, blocked the production of 9 biomass components when deleted. When amino acids were included, this effect dropped to 123 genes which blocked only 6 components on average. Even a relatively small amount of amino acids significantly improved the robustness of *B*. *fragilis*. This effect remained even when the maximum uptake of the amino acids was limited to 0.01 mmol/gDW/h. Table 3 identifies the genes that are no longer essential for growth with the addition of amino acids. There were six additional non-essential genes that decreased the growth rate by more than 10% when deleted in the glucose medium but not when supplemented with amino acids.

**Table 3:** Differentially Essential Genes.

Gene Identifier	Function	Metabolites Blocked
BF638R_RS02825	Aminotransferase	All 8 NTPs and dNTPs; NADH/NADPH; Several amino acids
BF638R_RS14560	Methionine synthase	6 amino acids
BF638R_RS19065	Methylenetetrahydrofolate reductase	Arginine, histidine, proline
BF638R_RS02840	Homoserine O-succinyltransferase	Arginine, histidine, proline

Each row in Table 3 corresponds to a gene in the model that is essential when grown on glucose as the sole carbon source but not when a small amount of amino acids is supplemented. The last column states which metabolites cannot be synthesized when that gene is deleted. One may expect that arginine, histidine, and proline production was restored by direct assimilation of those amino acids, but no transporter for these metabolites is currently annotated.

**Fig 5:**
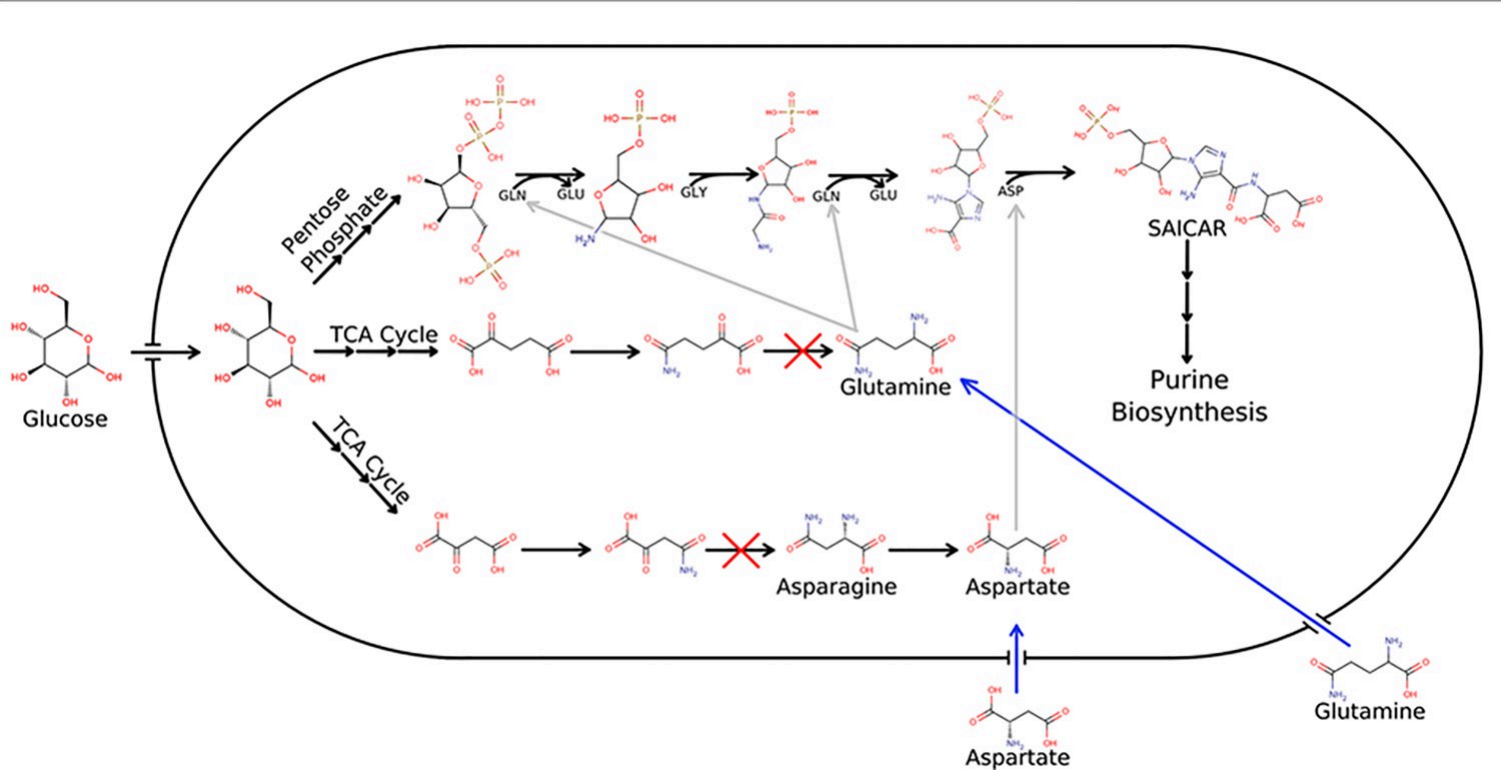
Purine biosynthesis is saved from gene knockouts by external amino acids. This figure shows an abbreviated view of purine synthesis in *B*. *fragilis*. The nitrogen in purine bases is taken from amino acids. These amino acids are produced in transamination reactions involving TCA intermediates. Thus, the gene knockouts corresponding to these transamination reactions (red Xs) are lethal, as the production of key amino acids and all purines is halted. However, if amino acids are made available in the environment (blue arrows), purine biosynthesis is able to proceed. Pyrimidine production is also aspartate dependent, so it is rescued similarly.

Fig 5 shows how the availability of amino acids saves nucleotide metabolism from the effects of deleterious knockouts.

No genes changed in essentiality when a mixture of fermentation products was supplemented to the model. While VFAs can supplement glucose by forming acyl-CoA, the cell is still dependent on glucose for most biomass precursors. VFAs and amino acids together allowed for low levels of growth (0.11 h ^-1^, 25% of that on glucose) without an additional carbon source. S5 Table contains the full results of these co-metabolism simulations.

Overall, these results suggest that even though amino acids and fermentation end products are neither necessary nor sufficient for biomass production alone, they provide an advantage for *B*. *fragilis*. Even with only minute influxes, amino acids allowed flux through several inactive pathways and allowed growth when otherwise essential genes were knocked out. The gut microbiome is a complex environment, so it is highly beneficial for *B*. *fragilis* to be able to utilize a wide range of metabolites. *B*. *fragilis*’ possible niches and metabolism within the colon could therefore be much broader than originally thought and might not only be limited to catabolism of polysaccharides.

### Human oligosaccharide catabolism in *B*. *fragilis*

The ability to digest HMOs is a major driving factor deciding which organisms are prevalent in the infant gut microbiome [26,29]. To further investigate the ability of *B*. *fragilis* to utilize components of human milk, reactions were added to represent the degradation of the 15 most abundant human milk oligosaccharides (HMOs) [30]. *B*. *fragilis* has been shown to digest some oligosaccharides in the periplasmic space [4], so these digestion reactions were assumed to be in the periplasm as well.

*B*. *fragilis* possesses enzymes capable of digesting a broad range of oligosaccharides. Each bond in each of these compounds could be paired to an enzyme potentially capable of breaking it. S6 Table provides a list of compounds and the enzymes needed to digest them. For several bonds, there are multiple enzymes in the genome that could perform the necessary hydrolysis. This aligns with previous work that suggests *B*. *fragilis* readily consumes HMOs [31] and bovine milk oligosaccharides [32] and suggests that *B*. *fragilis* could digest them completely. Other bacteria such as *Bifodobacterium* and *Enterococcus* spp. are nonetheless able to outcompete *B*. *fragilis* when certain HMOs are increased in abundance in the infant gut microbiome [33], indicating that the interplay between these microbes is defined by something other than whether or not the oligosaccharides can be utilized.

*B*. *fragilis* has been shown to be able to scavenge human N-linked glycans [4]. The ten most abundant N-linked glycans from human transferrin identified by Abu Bakar *et al* [34] were added as representatives. Again, all oligosaccharides were able to be fully divided into the component monomers in multiple ways. As human N-glycans share the same core and many repeated motifs, it is therefore likely that most such glycans can be used as a carbohydrate source for *B*. *fragilis*. The predicted ability to both liberate and consume the sialic acids in these oligosaccharides stands in contrast to the closely related *B*. *thetaiotaomicron*, which cannot consume the sialic acids it frees [35]. Sialic acids act as a carbon source for pathogenic *Clostridioides difficile* and *Salmonella typhimurium* [35], and have been shown in gnotobiotic mouse models to consume sialic acids liberated by *B*. *thetaiotaomicron* [36]. *B*. *fragilis* could therefore be competing with these pathogenic bacteria for sialic acid.

### Comparison of *i*MN674 to automated AGORA2 model

Heinken *et al* recently published semi-automatic reconstructions of 7,302 human associated microorganisms [37], including a model for *Bacteroides fragilis* 638R. This model, while refined through some experimentally available growth data, did not undergo the same degree of curation as *i*MN674. Excluding exchange reactions, the AGORA2 model has 936 reactions without genome annotation, compared to 9 reactions in our model. Among these 936 reactions, 103 are indispensable for the model to grow, even when all exchanges are fully open. This number increases to 133 essential genes with no annotations when limited to a glucose, ammonia, and mineral medium. None of the 621 transporters present in the AGORA2 model have gene associations. Overall, more than 50 percent of the non-exchange reactions lack genome annotations. *i*MN674, which has genome support for all but 9 reactions, contains a smaller reaction network, but the degree of certainty in these reactions is much higher. Moreover, *i*MN674 has been refined and curated to accurately reflect experimental data. When comparing model-data agreement as in Fig 3, our model achieves a sensitivity and specificity of 0.90 and 0.96; the AGORA2 model’s sensitivity and specificity are 0.55 and 0.88. While semi-automated models provide a good tool for the community, well curated models such as *i*MN674 contain an experimentally refined and evidenced reconstruction that provides the highest accuracy to study the metabolism of the target organism.

## Conclusion

Here we present the genome-scale metabolic model *i*MN674 of the ubiquitous gut bacterium *B*. *fragilis*. The model was refined and validated by examining the growth phenotypes of the bacterium identified in the literature and discovered via nutrient utilization experiments. In addition, the ATP requirements for growth and stasis were calculated to provide realistic growth rates. Lastly, the model was shown to be able to produce fermentation products in experimentally observed ratios.

The metabolic model allowed for the various fermentation products to be produced across a range of ratios at a fixed growth rate. This indicates that these ratios are controlled by some other feature of *B*. *fragilis*, such as transcriptional regulation or post-translational enzyme control. The fermentation yields of *B*. *fragilis* are influenced by the availability of carbohydrates and nitrogenous compounds in the media, highlighting the complex interplay between environmental conditions and metabolism for *B*. *fragilis*. The model demonstrates how even metabolites that cannot produce biomass by themselves can be co-metabolized by *B*. *fragilis* and could provide an ecological advantage for the bacteria to survive in diverse conditions.

Overall, we demonstrated that *i*MN674 accurately captures the metabolic nature of *B*. *fragilis* and acts as a platform for further hypothesis generation regarding the microorganism, the effects of our diet on its growth, and its influence on the host. The gaps in *i*MN674, in particular lack of knowledge about carbohydrate transporters, highlight gaps in our understanding of *B*. *fragilis*’ metabolism. Additional work is needed to further validate the model and contextualize its results. As this knowledge is acquired, it will be continually incorporated into *i*MN674, allowing it to continually improve its accuracy in quantitative phenotypic prediction.

## Methods

### Initial model generation

**Fig 6:**
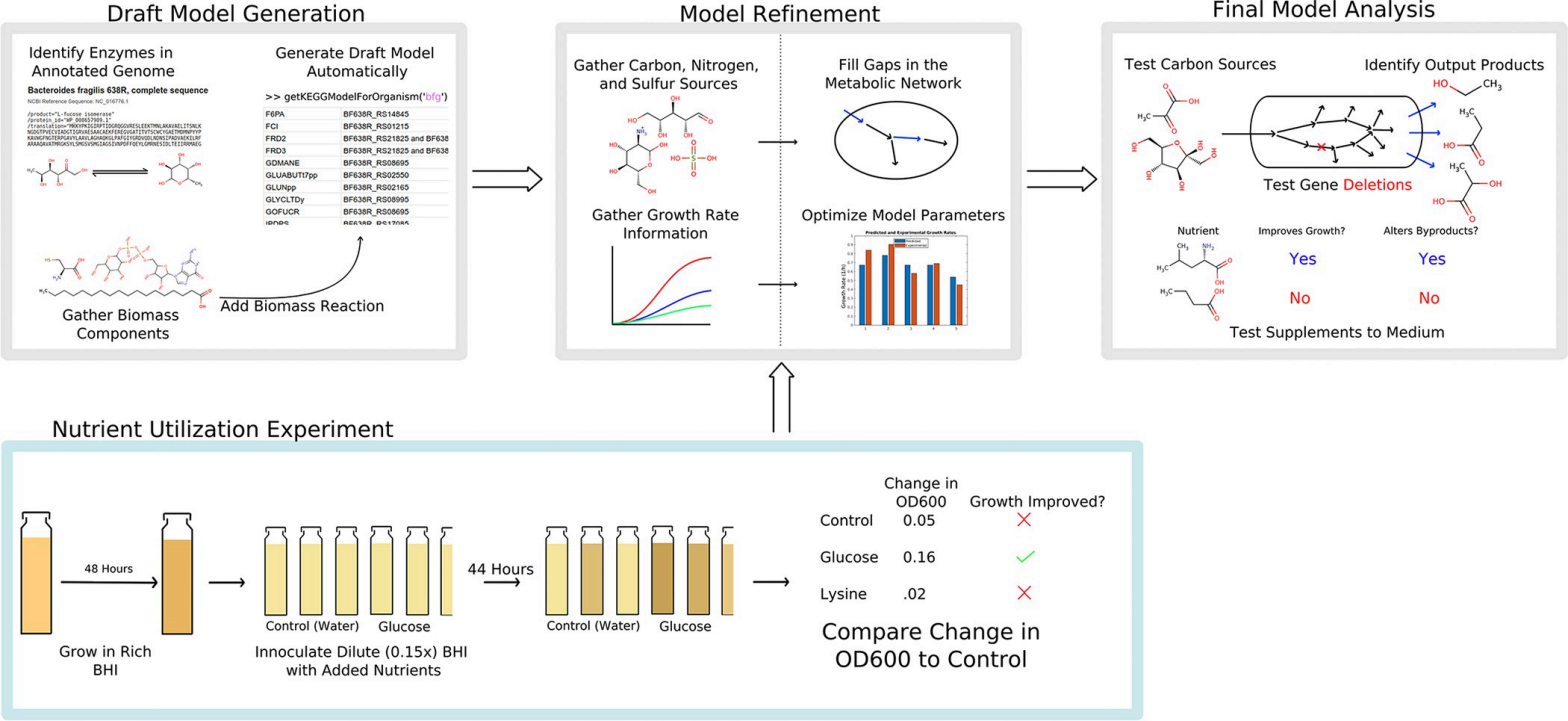
A flowchart outlining the general methodology from initial model generation to model analysis.

The initial draft models were generated using the RAVEN Toolbox version 2.5.0 (Fig 6, first panel. The rest of Fig 6 outlines the rest of the methods). [38]. A first draft was reconstructed via the KEGG species code method (getKEGGModelForOrganism(‘bfg’)), which generates a model based on the assigned protein homologies for the organism in question on KEGG (release 98) [39]. Another was made using a pre-trained hidden markov model to find homologous proteins in the organism’s genome (getKEGGModelForOrganism(’bfg’,bfg_’protein.faa’,’prok90_kegg94’). This was done using the same genome found for *B*. *fragilis* 638R (accession number GCA_000210835.1). The models made by either method were nearly identical and thus the gene-reaction associations from both were merged. These methods were chosen over using existing models as templates as few suitably phylogenetically related organisms with high-quality models could be found. Using KEGG allowed for a wider range of reactions to be considered, while template-based methods would only return the presumably limited set of reactions at the intersection of *B*. *fragilis’* and the template’s metabolism.

The RAVEN Toolbox’s output is in KEGG format. KEGG does not include transporters or detailed lipid reactions, so these were mapped to BiGG reactions (BiGG version 1.6). BiGG is a UC San Diego based database of GEMs in a mostly standardized format [12]. Additionally, uncommon reactions in *B*. *fragilis* not present in BiGG or KEGG had to be created and added manually, such as 2’-fucosyllactose degradation. Potential transporters and lipid reactions were identified by BLAST comparison to other gut bacteria models, including the *E*. *coli* model iML1515 [16] and the salmonella pan-reactome model iYS1720 [40]. Additional transporters were found in the Transporter Classification Database (2021 update) [41]. Custom reactions representing these transporters were created and added to the model. In the cases where *B*. *fragilis* is known to consume a metabolite but no suitable transporter could be found in the genome, the metabolite was assumed to enter the periplasm through porins then enter the cytosol.

The reactions were then manually curated. To be included in the model, a protein coding sequence from the genome must be sufficiently similar to a prokaryotic gene applied to that reaction in another model or in the KEGG enzyme database. Similarity was assessed by BLAST, with cutoffs of identity > 30%, query cover > 95%, and e-value < 10^−15^. Enzymes with multiple possible roles were initially assumed to have all the possible functions assigned to it in KEGG unless refuted by evidence found in the literature. For example, the gene 5’/3’-nucleotidase SurE has the enzyme commission numbers 3.1.3.5, 3.13.6, and 3.6.1.11 [42]. Each of these are associated with the hydrolysis of phosphate from a variety of nucleotides, leading SurE to be associated with 30 reactions. Reactions with sufficient BLAST scores were rejected if the genome annotation or other evidence suggested the genes associated with them were more likely to be associated with another, incompatible enzyme. Genes with vague annotations or unclear enzymatic function were noted as low confidence.

All metabolites in the model were paired with formulas and names. Complex cofactors that are not produced or consumed, but only cycled, were given the formula ‘R.’ These include tRNAs and acyl-carrier proteins. The metabolite formulas were kept consistent to prevent any reactions from producing or consuming any atoms except hydrogen. Molecule charge was not considered. Unbalanced or unclear reactions such as R08411 from KEGG were removed. All reactions were given descriptive names and subsystems. Reaction reversibility was assigned based on whether the majority of models in BiGG marked the reaction or similar reactions as reversible or not. All reversible reactions were given default bounds of +/- 1,000 mmol/gDW/h, while irreversible reactions had the lower bound set to 0 mmol/gDW/h. Exchange reactions were generally given bounds of [–20, 1,000] mmol/gDW/h.

### Biomass objective function

Data on the molecular composition of *B*. *fragilis* is limited. Consequently, data from a variety of sources had to be considered to construct an estimate of the biomass composition. The proportions of the organism’s dry weight in each major category (lipids, carbohydrates, proteins, DNA, and RNA) were taken from Frantz and McCallum (1980) [43], which examined strain ATCC 23745. The data from the exponential growth phase was averaged and used. The data did not sum to 100%, so the remainder was assumed to be the cofactor, mineral, and metabolite contribution not measured in the study.

Within the DNA category, the percent of each nucleotide was calculated by the percent of each found in the genome. Due to a lack of RNA-seq experiments, the relative amounts of each RNA monomer was assumed to be the same as in the genome.

Within the carbohydrates category, the amounts were taken from Cherniak *et al* [44] and Kasper [45] then averaged. These data were calculated from the outer membrane of various *B*. *fragilis* strains and were taken to be representative of the overall composition. As carbohydrate monomers are incorporated into polymers via nucleotide carriers, they are included in the biomass reaction in the form carrier-carbohydrate -> released carrier or as the consumption of polymerized monomers. This forced flux through the nucleotide-sugar reactions, improving the realism of the model and its energy needs.

The amino acid composition was found in Sok *et al* [46] for the related species *B*. *ruminicola*. This data did not measure the abundances of asparagine, glutamine, or tryptophan. The total did not add to 100%, so the unaccounted for abundance was divided equally between these. Similar to the carbohydrates, these metabolites were included in the form trna-amino acid -> free trna, with the amino acid being consumed.

The lipids were drawn from Eiichi and Miyagawa [47], which measured lipid composition in *B*. *fragilis*. Its results detailed the percent composition of each chain length and desaturation level, but not the headgroups. Reactions were added in the model to have at least one representative from each chain type. The percent biomass assigned to each chain type was divided equally between all the lipids present in that category in the model.

Lastly, cofactor, mineral, and vitamin consumption was taken from iML1515. The specific values were re-scaled to fit the metabolite and cofactor percentage used here. Some metabolites in this category were removed if they did not otherwise appear in the *B*. *fragilis* model and there was no experimental evidence to expect they were a necessary component. The final biomass objective function was calculated by normalizing the sum of weights of all these molecules to be 1 gram. For metabolites with carrier molecules, the mass used in the calculation was that of the free metabolite.

### Gap filling

The draft model could not produce every biomass precursor. To fill these gaps, new reactions were added from BiGG and KEGG. The model was then run with all exchange reactions open and optimized for maximal production of each biomass metabolite. If a given reaction improved the number of biomass components that could be produced, it was appended to a list of new reactions to confirm via BLAST or literature search. This was repeated iteratively until the model could produce all precursors and therefore yield a non-zero flux through the biomass reaction. The process was repeated until the model could grow under all conditions found in the literature (see S7 Table).

The draft model contained many reactions that could not carry flux. Many of these were secondary uses of enzymes. Such enzymes are a major source of dead-end metabolites and blocked reactions in GEMs [10,48]. Some reactions, including transporters of common metabolites and fragments of common pathways, could be connected to the rest of the metabolic network by gap filling. 399 reactions remain that cannot carry flux. 87 of them have dead end metabolites on both ends, leaving them entirely disconnected from the network.

### ATP maintenance coefficients

The ATP maintenance coefficients related to growth and stasis (GAM and NGAM) were calculated by minimizing the error between growth rates found in the literature and predicted by the model. These included the growth rates shown in Varel and Bryant [20], Fig 1, and Spence et al [19] Fig 2A and 2B. Where peptides or casitone were mentioned, we activated all amino acid exchange reactions. S4 Table details the medium compositions for this process under Fig 3, but generally exchanges for main carbon and nitrogen sources were set to 20 mmol/gDW/h. This process is implemented in the script for Fig 3. At each iteration, the model was optimized in each condition and the growth rates were recorded. The overall error was defined as the maximum relative error between the model and experimental values, as to constrain the largest error. The error was then minimized using MATLAB’s fminsearch function with an initial estimation of [100,6]. This yielded a GAM and NGAM of 24.9 and 38.9. Repeating with a grid of initial guesses always returned these values.

### Nutrient utilization experiment

*Bacteroides fragilis* 638R was grown from a glycerol stock in 50 mL of anaerobic BHI media (Teknova B9500). This media was supplemented with a 1% dilution of Remel R450951 vitamin K and hemin solution, 4 mM cysteine, 0.1% weight by volume yeast extract (RPI Y20020-250), and a 1% dilution of trace minerals (ATCC MD-TMS), and a pinch of resazurin. The bacteria were grown for two days before the experiment to reach exponential phase. Two liters of the same media but at 0.15x BHI concentration were mixed, degassed, and autoclaved as well in sealed anaerobic bottles. 0.15x BHI was selected to give low but non-zero growth, so that improvements from the added nutrient would be more visible.

Autoclaved 16x125mm Hungate tubes with butyl stoppers were used to test the individual conditions. The tubes were individually opened, then a needle with flowing nitrogen placed into it to displace oxygen and provide positive pressure. Similar needles were placed into the 0.15x BHI media bottles after opening to ensure they remained anoxic. Using a serological pipette, 10ml of the media was placed in each tube before sealing. The tubes were allowed to rest until the color of resazurin dissipated. Then 100 microliters of a prepared 0.5 M solution of one of the nutrients was added, bringing the tube to 5 mM. Lastly, 100 microliters of the growing bacteria were added. This was repeated for each nutrient in triplicate. To the control tube 100 microliters of water was added in place of a nutrient.

200 microliters were drawn from each tube for an initial OD600 reading, and then the tubes were incubated at 37 C for 44 hours before taking a final OD600 reading. To decide if a set of tubes showed growth, the average change in OD600 for that condition was compared to the average OD600 change of the control tubes. If it was more than 0.05 higher, the condition was considered to have shown improved growth over the control. Otherwise it was considered to have shown no improvement.

The gap filling was then repeated to allow for growth under any newly identified conditions. In the model, a growth increase of 0.01 or higher was considered to be a positive result. If the model predicted growth where the data suggested it should not, low confidence reactions and transporters were removed to eliminate the false positive.

### Computation

All simulations were performed in MATLAB R2022b on an UBUNTU version 22.04.1 workstation. Optimizations were performed using the flux balance analysis feature of the Cobra Toolbox (version 3.4) [49] using the solver Gurobi 9.5.2. Chemical structures were generated using the Smi2Depict web interface of ChemDB [50]. FBC curation to make a standard reference for *i*MN674 was made using FROG analysis software (version 0.2.2) [51]. A MEMOTE report for the model was generated with MEMOTE 0.11.1 (S3 File) [52].

## Supporting information

S1 Table Essential Genes. (XLSX)

S2 Table Reactions Added Without Genome Annotation. (XLSX)

S3 Table Nutrient Utilization Experiment and Model Agreement. (XLSX)

S4 Table *In Silico* Media Conditions. (XLSX)

S5 Table Supplementary Nutrients for Growth and VFA Production. Sheet 1 in this file shows how the growth on each exchange reaction in the model was altered by the addition of a fermentation product mixture, amino acid mixture, or both. Sheet 2 shows how different supplementary metabolites alter the potential for VFA production.
(XLSX)

S6 Table Enzymes Utilized in Human Oligosaccharide Degradation. (XLSX)

S7 Table Media Conditions from Literature. This lists semi-or fully defined media conditions found in literature used in gap-filling the model.
(XLSX)

S1 File *i*MN674 in various Formats. This is the *i*MN674 model presented in.mat, COBRApy.json, and SBML.xml formats, set to model a glucose and mineral medium.
(ZIP)

S2 File *i*MN674 in Spreadsheet Format. (XLSX)

S3 File MEMOTE Report. (PDF)

S4 File FROG Report. This file contains the FBC curation of the model to present a reproducible model state for model behavior validation across systems. It also reports the effects of gene and reaction deletions in the provided medium.
(ZIP)

S5 File Figure Code. These files contain the code needed to reproduce the figures.
(ZIP)

S6 File Nutrient Utilization Raw Data. These show the initial and final OD600 values found in the bacterial growth experiment.
(XLSX)
