# Supplementary material for: Genome-scale metabolic modeling of the human gut bacterium Bacteroides fragilis strain 638R
Source: PLoS Comput Biol. 2023 Oct 30;19(10):e1011594. doi: 10.1371/journal.pcbi.1011594 (PMC10635569; doi:10.1371/journal.pcbi.1011594)
Supplement: S3 File — (PDF) [file pcbi.1011594.s010.pdf]

Independent Section

Contains tests that are independent of the class of modeled organism, a model's complexity or types of identifiers that are used to describe its components. Parameterization or initialization of the network is not required. See readme for more details.

Consistency

Stoichiometric Consistency	0.0% (x3)
Mass Balance	70.6%
Charge Balance	100.0%
Metabolite Connectivity	100.0%
Unbounded Flux In Default Medium	48.7%
Sub Total	46% (x3)

Annotation - Metabolites

Presence of Metabolite Annotation	64.8%
Metabolite Annotations Per Database	Info
pubchem.compound	0.0%
kegg.compound	54.6%
seed.compound	59.3%
inchikey	0.0%
inchi	0.0%
chebi	56.9%
hmdb	44.1%
reactome	0.0%

Specific Section

Covers general statistics and specific aspects of a metabolic network that are not universally applicable. See readme for more details.

SBML

SBML Level and Version	SBML Level 3 Version 1
FBC enabled	true

Basic Information

Model Identifier	model
Total Metabolites	1,362
Total Reactions	1,634
Total Genes	677
Total Compartments	4
Metabolic Coverage	2.41
Uncoserved Metabolites	3
Minimal Inconsistent Net Stoichiometries	3

Metabolite Information

Unique Metabolites	1,109
Duplicate Metabolites in Identical Compartments	0
Metabolites without Charge	0
Metabolites without Formula	0

reactome	0.0%
metanetx.chemical	64.4%
bigg.metabolite	64.8%
biocyc	57.4%
Metabolite Annotation Conformity Per Database	Info
pubchem.compound	0.0%
kegg.compound	100.0%
seed.compound	100.0%
inchikey	0.0%
inchi	0.0%
chebi	100.0%
hmdb	100.0%
reactome	0.0%
metanetx.chemical	100.0%
bigg.metabolite	100.0%
biocyc	100.0%
Uniform Metabolite Identifier Namespace	99.1%

Sub Total	66%

Annotation - Reactions

Presence of Reaction Annotation	56.4%
Reaction Annotations Per Database	Info
rhea	31.9%

Metabolites without Formula	[illegible]
Medium Components	12

Reaction Information

Purely Metabolic Reactions	1,196
Purely Metabolic Reactions with Constraints	13
Transport Reactions	288
Transport Reactions with Constraints	1
Reactions With Partially Identical Annotations	0.14
Duplicate Reactions	0.00
Reactions With Identical Genes	0.80

Gene-Protein-Reaction (GPR) Associations

Reactions without GPR	0
Fraction of Transport Reactions without GPR	0.00
Enzyme Complexes	77

Biomass

Biomass Reactions Identified	6
Biomass Consistency	Info
BIOMASS	Errored
BIOMASS_AMINO_ACIDS	Errored
BIOMASS_CARBOHYDRATES	Errored
BIOMASS_LIPIDS	Errored

Annotation - Reactions

Presence of Reaction Annotation	56.4%
Reaction Annotations Per Database	Info
rhea	31.9%
kegg.reaction	25.8%
seed.reaction	45.1%
metanetx.reaction	55.4%
bigg.reaction	56.4%
reactome	0.0%
ec-code	33.8%
brenda	0.0%
biocyc	32.3%
Reaction Annotation Conformity Per Database	Info
rhea	99.4%
kegg.reaction	100.0%
seed.reaction	100.0%
metanetx.reaction	100.0%
bigg.reaction	100.0%
reactome	0.0%
ec-code	99.0%
brenda	0.0%
biocyc	100.0%
Uniform Reaction Identifier Namespace	98.5%

BIOMASS	Errored
BIOMASS_AMINO_ACIDS	Errored
BIOMASS_CARBOHYDRATES	Errored
BIOMASS_LIPIDS	Errored
BIOMASS_MISC	Errored
BIOMASS_NUCLEIC_ACIDS	Errored
Biomass Production In Default Medium	Info
BIOMASS	0.43
BIOMASS_AMINO_ACIDS	0.43
BIOMASS_CARBOHYDRATES	0.43
BIOMASS_LIPIDS	0.43
BIOMASS_MISC	0.43
BIOMASS_NUCLEIC_ACIDS	0.43
Unrealistic Growth Rate In Default Medium	Info
BIOMASS	false
BIOMASS_AMINO_ACIDS	false
BIOMASS_CARBOHYDRATES	false
BIOMASS_LIPIDS	false
BIOMASS_MISC	false
BIOMASS_NUCLEIC_ACIDS	false
Biomass Production In Complete Medium	Info
BIOMASS	134.00
BIOMASS_AMINO_ACIDS	134.00

biocyc	100.0%	
Uniform Reaction Identifier Namespace	98.5%	
Sub Total	66%	

Annotation - Genes

Presence of Gene Annotation	99.6%	
Gene Annotations Per Database	Info	
refseq	0.0%	
uniprot	0.0%	
ecogene	0.0%	
kegg.genes	99.6%	
ncbigi	0.0%	
ncbigene	99.6%	
ncbiprotein	0.0%	
ccds	0.0%	
hprd	0.0%	
asap	0.0%	
Gene Annotation Conformity Per Database	Info	
refseq	0.0%	
uniprot	0.0%	
ecogene	0.0%	
kegg.genes	100.0%	
ncbigi	0.0%	

BIOMASS	134.00	
BIOMASS_AMINO_ACIDS	134.00	
BIOMASS_CARBOHYDRATES	134.00	
BIOMASS_LIPIDS	134.00	
BIOMASS_MISC	134.00	
BIOMASS_NUCLEIC_ACIDS	134.00	
Blocked Biomass Precursors In Default Medium	Info	
BIOMASS	0	
BIOMASS_AMINO_ACIDS	20	
BIOMASS_CARBOHYDRATES	0	
BIOMASS_LIPIDS	0	
BIOMASS_MISC	0	
BIOMASS_NUCLEIC_ACIDS	0	
Blocked Biomass Precursors In Complete Medium	Info	
BIOMASS	0	
BIOMASS_AMINO_ACIDS	20	
BIOMASS_CARBOHYDRATES	0	
BIOMASS_LIPIDS	0	
BIOMASS_MISC	0	
BIOMASS_NUCLEIC_ACIDS	0	
Ratio of Direct Metabolites in Biomass Reaction	Info	
BIOMASS	0.00	
BIOMASS_AMINO_ACIDS	0.00	

ncbigi	0.0%
ncbigene	0.4%
ncbiprotein	0.0%
ccds	0.0%
hprd	0.0%
asap	0.0%
Sub Total	43%

Annotation - SBO Terms

Metabolite General SBO Presence	64.8%
Metabolite SBO:0000247 Presence	64.8%
Reaction General SBO Presence	56.4%
Metabolic Reaction SBO:0000176 Presence	48.8%
Transport Reaction SBO:0000185 Presence	78.1%
Exchange Reaction SBO:0000627 Presence	76.8%
Demand Reaction SBO:0000628 Presence	100.0%
Sink Reactions SBO:0000632 Presence	Skipped
Gene General SBO Presence	0.0%
Gene SBO:0000243 Presence	0.0%
Biomass Reactions SBO:0000629 Presence	0.0%
Sub Total	45% x2
Total Score	48%

BIOMASS_AMINO_ACIDS	0.00
BIOMASS_CARBOHYDRATES	0.00
BIOMASS_LIPIDS	0.37
BIOMASS_MISC	0.33
BIOMASS_NUCLEIC_ACIDS	0.00
Number of Missing Essential Biomass Precursors	Info
BIOMASS	26
BIOMASS_AMINO_ACIDS	37
BIOMASS_CARBOHYDRATES	32
BIOMASS_LIPIDS	37
BIOMASS_MISC	33
BIOMASS_NUCLEIC_ACIDS	3

Energy Metabolism

Non-Growth Associated Maintenance Reaction	0
Growth-associated Maintenance in Biomass Reaction	Info
BIOMASS	false
BIOMASS_AMINO_ACIDS	false
BIOMASS_CARBOHYDRATES	false
BIOMASS_LIPIDS	false
BIOMASS_MISC	false
BIOMASS_NUCLEIC_ACIDS	false
Number of Reversible Oxygen-Containing Reactions	10

Biomass Reactions SBO:0000629 Presence	0.0%	
Sub Total	45%	
Total Score	48%	

Total Score

48%

Score per Category

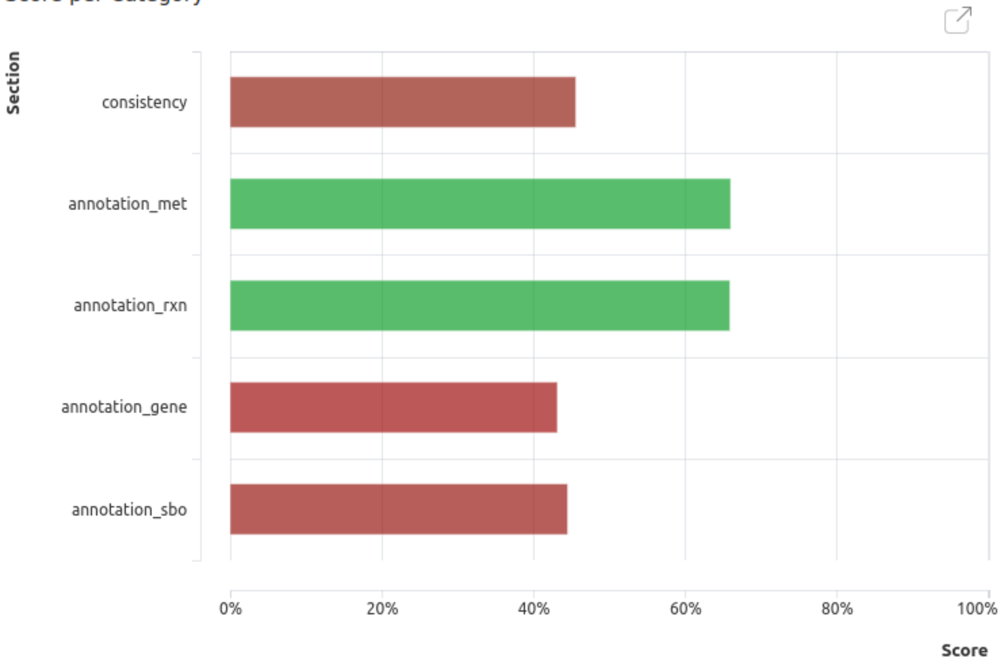

BIOMASS_LIPIDS	false
BIOMASS_MISC	false
BIOMASS_NUCLEIC_ACIDS	false
Number of Reversible Oxygen-Containing Reactions	10
Erroneous Energy-generating Cycles	Info
MNXM3	Skipped
MNXM63	Skipped
MNXM51	Skipped
MNXM121	Skipped
MNXM423	Skipped
MNXM6	Skipped
MNXM10	Skipped
MNXM38	Skipped
MNXM208	Skipped
MNXM191	Skipped
MNXM223	Skipped
MNXM7517	Skipped
MNXM12233	Skipped
MNXM558	Skipped
MNXM21	Skipped
MNXM89557	Skipped

Network Topology

[illegible]

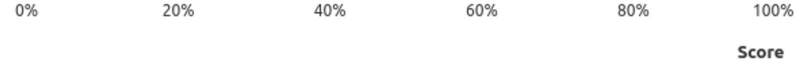

Network Topology

Universally Blocked Reactions	399
Orphan Metabolites	86
Dead-end Metabolites	68
Stoichiometrically Balanced Cycles	419
Metabolite Production In Complete Medium	610
Metabolite Consumption In Complete Medium	653

Matrix Conditioning

Ratio Min/Max Non-Zero Coefficients	0.00
Independent Conservation Relations	189
Rank	1173
Degrees Of Freedom	461

Experimental Data Comparison

Growth Prediction	Skipped
Gene Essentiality Prediction	Skipped

Misc. Tests

Environment

Python Version	3.10.12
Platform	Linux
Memote Version	0.13.0
